# Supplementary material for: Perioperative Techniques for the Use of Botulinum Toxin in Overactive Bladder: Results of a Multinational Online Survey of Urogynecologists in Germany, Austria, and Switzerland
Source: J Clin Med. 2023 Feb 12;12(4):1462. doi: 10.3390/jcm12041462 (PMC9964329; doi:10.3390/jcm12041462)
Supplement: Supplementary file 1 [file jcm-12-01462-s001.zip › jcm-2182477-supplementary.pdf]

Supplementary Material
Survey: “Survey about the use of botulinumtoxin injections for the therapy of overactive bladder and mixed-urinary incontinence”
(translation from German in English)

1. Do you use botulinumtoxin injections for the treatment of overactive bladder and/or mixed urinary incontinence?
    - Yes
    - No

2. For which type of urinary incontinence do you use botulinumtoxin injections? (multiple answers allowed)
    - idiopathic overactive bladder
    - neurogenic overactive bladder
    - mixed urinary incontinence with dominant overactive bladder
    - other indications

3. When do you use botulinumtoxin injections as a therapy?
    - as a first-line therapy (prior to conservative and/or oral drug therapy)
    - as a second-line therapy (after failure of oral drug therapy)
    - as a third-line therapy (after failure of pudendal nerve stimulation or sacral neuromodulation.

4. Do you perform the treatment as an inpatient and outpatient treatment (please do not account for inpatient treatment due to treatment complications, but only planned treatments)?
    - only inpatient
    - only outpatient
    - inpatient and outpatient

5. Where do you perform the procedure?
    - in the operation room (inpatient and outpatient operation room)
    - in the outpatient clinic

6. Which type of product do you use? (multiple answers allowed)
    - Botox®
    - Dysport®
    - Xeomin®
    - NeuroBloc®
    - other product: __________________ (please indicate the product)

7. How many units of Botox®/Xeomin® do you use in the primary situation?
    - 50
    - 100
    - 150
    - 200
    - >200

8. How many units of Dysport® do you use in the primary situation?
    - <100
    - 100
    - 200
    - 300
    - 400
    - > 400

9. How many units of NeuroBloc® do you use in the primary situation?
    - 5.000
    - 10.000
    - 15.000
    - 20.000
    - >20.000

10. Which type of anesthesia do you use?
    - anesthesia applied by anesthesiologist (i.e., sedation, general anesthesia, locoregional anesthesia)
    - intravesical local anesthesia
    - no anesthesia

11. Which type of anesthesia is applied by the anesthesiologist? (multiple answers allowed)
    - general anesthesia
    - sedation
    - spinal anesthesia

12. Do you apply preoperative antibiotics?
    - yes
    - no
    - case-by-case decision

13. Which type of cystoscope do you use? (multiple answers allowed)
    - rigid cystoscope
    - flexible cystoscope
    - other type: ________________ (please indicate)

14. How many injections do you apply in general?
    - 0 – 10
    - 11 – 20
    - 21 – 30
    - 31 – 40
    - 41 – 50
    - > 50

15. How many milliliters do you apply with each single injection?
    - 0.1 – 0.5 ml
    - 0.6 – 1.0 ml

- 1.1 – 1.5 ml
- 1.6 – 2.0 ml
- > 2.0 ml

16. Do you use needles with markings to evaluate depth of injection?
    - yes
    - no

17. Do you spare the ureteric ostia and/or the trigone from injections?
    - yes
    - no
    - case-by-case decision
    -

18. Which is your preferred site of injection? (multiple answers allowed)
    - posterior bladder wall
    - lateral bladder wall
    - bladder apex
    - trigone

19. Do you insert an indwelling catheter at the end of the procedure?
    - yes
    - no

20. Do patients receive a perioperative teaching on how to perform intermittent self-catheterization?
    - yes
    - no

21. Do you control the postvoid volume after the procedure?
    - yes
    - no

22. When do you measure the postvoid volume? (multiple answers allowed)
    - at the day of the procedure or prior to patient's discharge, resp.
    - in the first 7 days after the procedure
    - in the first 4 weeks after procedure
    - later then 1 month after the procedure

23. Do you repeat the procedure in case of treatment failure?
    - yes
    - no

24. In the first 12 months after the procedure, when do you repeat the procedure for the first time in case of treatment failure? (multiple answers allowed)
    - in the first 4 weeks
    - in months 1 – 3
    - in months 3 – 6
    - in months 6 – 12

- never in the first year
- decision is left to the patient

25. What is your maximum number of treatment repetitions per patient?
    - one
    - two
    - three
    - no limit

26. Where do you work? (multiple answers allowed)
    - university hospital
    - teaching hospital
    - community hospital
    - outpatient clinic
    - other type: ____________________ (please indicate)

27. In which of the following structures du you work?
    - certified pelvic floor center
    - urogynecologic unit without certification
    - general gynecologic unit without urogynecologic unit

28. How often do you perform this type of procedure per year?
    - 1 – 5 times
    - 6 – 10 times
    - 11 – 15 times
    - 16 – 20 times
    - > 20 times

29. What is your level of training?
    - resident
    - registrar
    - consultant
    - fellow in urogynecology

30. Do you have a board-certification in urogynecology (AGUB I – III in Germany, AUB module in Austria, or clinical fellowship in urogynecology in Switzerland) ?
    - yes
    - no

31. You declare yourself:
    - male
    - female
    - divers

32. Since how many years do you work in your specialty?
    - 0 – 5 years
    - 6 – 10 years
    - 11 – 20 years
    - > 20 years
